# The ischemic area-at-risk on T2-weighted MRI shows recovery of systolic strain at 1 year

**DOI:** 10.1186/1532-429X-13-S1-P90

**Published:** 2011-02-02

**Authors:** Declan P O'Regan, Ben Ariff, Clare Neuwirth, Yvonne Tan, Giuliana Durighel, Stuart A Cook

**Affiliations:** 1 Imperial College, London, UK

## Introduction

Animal models have demonstrated a recovery in systolic strain following reperfusion of acutely ischemic myocardium, however this has not been shown in clinical practice.

## Purpose

We aimed to determine whether there was long term improvement of regional strain in acutely ischemic myocardium following primary coronary intervention using cardiac magnetic resonance imaging (CMR)

## Methods

We studied 16 patients with acute ST elevation myocardial infarction (STEMI) who had received successful primary coronary intervention (PCI) to the culprit coronary artery. CMR was performed on a 1.5T Philips Achieva system (Best, Netherlands) within 7 days of PCI and again at 1 year. We used segmentation of T2-weighted spectrally-selective inversion recovery (SPIR) imaging to detect the ischemic area-at-risk and delayed enhancement inversion recovery imaging to measure infarct size and transmural thickness. Complementary spatial modulation of magnetization (CSPAMM) tagging with harmonic phase analysis (HARP) was used to measure peak midwall systolic strain at baseline and follow-up. Each section was divided according to the American Heart Association classification providing a total of 96 myocardial segments for analysis at each visit.

## Results

All patients showed acute myocardial edema within the reperfused territory and a variable transmural extent of enhancing necrosis. Mean myocardial salvage, given as the proportion of viable myocardium within the ischemic area-at-risk, was 62±21% at baseline. The mean infarct area at baseline was 20±10.2% and 14±5.7% at 1 year follow-up. In total 45 segments showed enhancing necrosis and the peak systolic midwall circumferential strain (Ecc) improved between baseline and follow-up ( -14.4±5.3% vs -19.2±5.4%, p<0.0001). In the remaining non-infarcted 45 segments there was no change in Ecc (-18.7±6.8% vs -21.1±6.1%, NS). The mean radial transmural extent of necrosis showed no correlation with ΔEcc (r^2^=0.01, NS).

## Conclusions

These findings demonstrate a long-term improvement in systolic function in acutely ischemic reperfused myocardium.

